# Prevention of urinary catheter-associated infections by coating antimicrobial peptides from crowberry endophytes

**DOI:** 10.1038/s41598-019-47108-5

**Published:** 2019-07-24

**Authors:** Claudia Monteiro, Fabíola Costa, Anna Maria Pirttilä, Mysore V. Tejesvi, M. Cristina L. Martins

**Affiliations:** 1 i3S, Instituto de Investigação e Inovação em Saúde, Universidade do Porto, Porto, Portugal; 2 INEB, Instituto de Engenharia Biomédica, Universidade do Porto, Rua Alfredo Allen, 208, 4200-135 Porto, Portugal; 3 Department of Ecology and Genetics, University of Oulu, Oulu, Finland; 4 CHAIN ANTIMICROBIALS ltd, Teknologiantie 2, Oulu, 90590 Finland; 5 Instituto de Ciências Biomédicas Abel Salazar, Universidade do Porto, Porto, Portugal

**Keywords:** Bacterial infection, Antibiotics

## Abstract

Urinary catheters are extensively used in hospitals, being responsible for about 75% of hospital-acquired infections. In this work, a *de novo* designed antimicrobial peptide (AMP) Chain201D was studied in the context of urinary catheter-associated infections. Chain201D showed excellent antimicrobial activity against relevant ATCC strains and clinical isolates of bacteria and yeast and demonstrated high stability in a wide range of temperatures, pH and salt concentrations. Moreover, the bactericidal activity of Chain201D immobilized on a model surface was studied against *Escherichia coli* (*E*. *coli*) and *Staphylococcus aureus* (*S*. *aureus*), some of the most prevalent strains found in urinary catheter-associated infections. Chain201D was successfully tethered to ((1-mercapto-11-undecyl)-(tetra(ethylene glycol) (EG4)) terminated self-assembled monolayers (SAMs), (EG4-SAMs), activated by 1,1′-Carbonyldiimidazole (CDI) at different concentrations. Chain201D surfaces can bind and kill by contact a high percentage of adherent bacteria. These achievements are obtained without any peptide modification (for chemoselective conjugation) and without the use of a spacer. Moreover, increased amounts of immobilized AMP lead to higher numbers of adhered/dead bacteria, revealing a concentration-dependent behaviour and demonstrating that Chain201D has excellent potential for developing antimicrobial urinary catheters.

## Introduction

Bacterial resistance is becoming a significant threat to public health, and it is estimated that a failure to address this problem would result in 10 million deaths every year globally by 2050^1,2^. Antibiotic resistance is particularly problematic in the hospital environment, being the cause of a large number of nosocomial infections^3,4^. The use of indwelling medical devices represents one of the significant risk factors for acquiring a nosocomial infection, as the materials used are still prone to bacterial adhesion, proliferation, biofilm formation and consequently infection^5^. Medical devices such as catheters, ventilators, implantable devices and surgical materials are responsible for the onset of a large variety of infections^6^. The use of urinary catheters alone has been reported to be responsible for about 75% of nosocomial infections^7^. Several gram-positive (*Staphylococcus* spp, *Streptococcus* spp and *Enterococcus* spp), gram-negative bacteria (*E*. *coli*, *Klebsiella pneumonia (K*. *pneumonia)*, *Pseudomonas* spp. and *Acinetobacter baumannie (A*. *baumannie*)) and yeast (*Candida* spp.) have been found associated with this type of infections^8–10^. Among these, *E*. *coli* has been the most prevalent strain found in urinary catheter-associated infections^11^. This microorganism has long been resistant to TEM-1 β-lactamase and, more recently, has become resistant to extended-spectrum β-lactamases (ESBLs), carbapenemases or acquired plasmid-mediated quinolone resistance (PMQR)^11^. Considering this scenario, it is urgent to find alternatives to conventional antibiotics that can prevent/treat infections associated with urinary catheters.

AMPs have been explored to improve the antimicrobial efficacy of medical devices, and currently are one of the most promising alternatives to conventional antibiotics^12–16^. AMPs are ubiquitous and no widespread resistance has been detected, making them excellent candidates for a novel class of antibiotics^17,18^. In addition to their bactericidal activity, many innate AMPs are also modulators of the immune system exerting their antimicrobial activity by several mechanisms of action^19^. Most AMPs have a broad spectrum of activity, being effective against gram-positive and gram-negative bacteria, fungi and viruses, being therefore suitable for polymicrobial infection scenarios. However, most AMPs have failed to enter clinical trials, as their development has been hampered by drawbacks such as reduced activity due to salt, serum, and pH sensitivity, susceptibility to proteolysis and peptide aggregation^20,21^.

From an expressed microbiome library of crowberry (*Empetrum nigrum*), we earlier identified a novel protein En-MAP1^22^. The protein showed antimicrobial activity when expressed as a recombinant protein in an *E*. *coli* host and fragmented^22^. One of the peptides that resulted from En-AP1 fragmentation, Met11, had a moderate antimicrobial activity and was chosen as a template for rationally designing of a *de novo* AMP, which was then synthesized from unnatural D-amino acids (D-AA). In most cases, D-AA-containing AMPs display the same antimicrobial activity while exhibiting considerably better selective toxicity than their L-AA enantiomeric counterpart^23^. Also, D-AA-containing AMPs are less prone to degradation and tend to be more stable towards proteolysis^23^. The developed AMP was named Chain201D (WO2017001731A1)^24^.

In recent years, covalent immobilization of AMPs on a surface has been used as a strategy to provide antimicrobial properties to medical devices preventing the need for systemic treatment. It has been previously shown that under certain conditions AMPs can maintain their antimicrobial activity when immobilized to different substrates^12–14,25^. This strategy provides a higher AMP surface availability and a more homogeneous distribution over the surface than peptide incorporation or adsorption methods, where peptide aggregation or uneven distribution usually occurs^26^. Moreover, peptide covalent immobilization has been pointed out as a solution to overcome enzymatic degradation, therefore increasing long-term stability and avoiding toxicity associated with the application of high AMP concentrations^27,28^.

Self-assembled monolayers (SAMs) have been used as a model system to study the effects of AMP immobilization on antimicrobial properties and underlying mechanisms of action, as SAMs are well-organized organic surfaces, which allows control of the surface at the molecular level and are easy to produce and functionalize^29,30^. Studies based on AMP-modified SAMs are of most value for the rational design of AMP-based antimicrobial surfaces, namely coatings for medical devices or even nanoparticles for targeting therapies.

**Figure 1:**
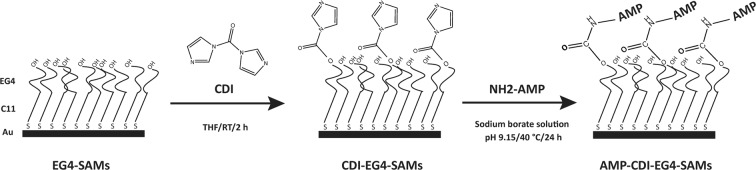
Schematic representation of Chain201D immobilization on EG4-SAMs.

In this work, we intended to assess the potential antimicrobial activity of Chain201D in the scope of urinary catheter-associated infections and when immobilized on surfaces as a proof of concept for a possible application on coatings for urinary catheters. Chain201D was first tested using several strains of bacteria and yeast relevant in urinary catheter-associated infections in a variety of conditions regarding temperature, pH and salt concentrations. Then Chain201D was immobilized on EG4 SAMs previously activated by CDI (Fig. 1) and further tested for antimicrobial activity against *E*. *coli* and *S*. *aureus*. Immobilization of a non-active peptide (CEM7) with similar length, positive charge and hydrophobicity than Chain201D was also performed as a control. CEM7 is a 12-mer derivative of MSI-78 (22-mer) that did not show antimicrobial activity in solution against several microorganisms, including the strains used in this study^31^.

## Results

### Designing peptide Chain201D, antimicrobial activity and stability tests

The peptide Chain201D (KWIVWRWRFKR) was designed by changing amino acids such as tryptophan (W), arginine (R), phenylalanine (F), and lysine (K) at various positions of the initial peptide Met11 (NRIVQQRTSSR). Modifications were done to improve antimicrobial activity according to various parameters, such as positive charge, aliphatic index, and instability index^32,33^. The antimicrobial activity of Chain201D was tested by using minimal inhibitory concentration (MIC) assays against *E*. *coli* (ATCC 25922), *S*. *aureus* (ATCC 25923), *K*. *pneumoniae* (ATCC 10031) and *Pseudomonas aeruginosa* (*P*. *aeruginosa*) (ATCC 27853). The MIC values were 2 and 4 ug/ml for *E*. *coli and S*. *aureus*, respectively and 8 ug/ml for *K*. *pneumoniae* and *P*. *aeruginosa* (Table 1).

**Table 1:** MIC (μg/mL) of peptide Chain201D tested against ATCC strains as determined by the microdilution method.

ATCC strains	MIC (μg/mL)
*E*. *coli* (ATCC 25922)	2
*S*. *aureus* (ATCC25923)	4
*K*. *pneumoniae* (ATCC 10031)	8
*P*. *aeruginosa* (ATCC 27853)	8

Chain201D was very active against the four ATCC strains tested, and therefore, we then tested it against five clinical strains per species (*E*. *coli*, *K*. *pneumoniae*, *Enterobacter cloacae* (*E*. *cloacae*), *P*. *aeruginosa*, *Streptococcus pneumoniae* (*S*. *pneumoniae*), *A*. *baumannii* and *S*. *aureus*), for which the MIC are presented in Table 2, and four clinical strains of yeasts including *Candida albicans* (*C*. *albicans*), *Candida glabrata* (*C*. *glabrata*) and *Candida parapsilosis* (*C*. *parapsilosis*) (Table 2). Results confirm the broad spectrum of activity of Chain201D against gram-negative and gram-positive bacteria and fungus.

**Table 2:** MIC (μg/mL) of peptide Chain201D tested against clinical isolates of bacteria and yeast (1–5) as determined by the microdilution method.

Bacterial clinical isolates	1	2	3	4	5
*E*. *coli*	16	1	1	1	1
*K*. *pneumonia*	2	2	4	4	2
*P*. *aeruginosa*	4	4	4	2	2
*E*. *cloacae*	1	2	2	2	1
*S*. *aureus*	1	1	1	1	1
*S*. *pneumonia*	8	4	2	4	4
*A*. *baumannii*	4	4	4	4	4
Yeast clinical isolates	1	2	3	4	5
*C*. *albicans*	16	8	8	16	8
*C*. *glabrata*	8	8	8	64	
*C*. *parapsilosis*	8	8	8	8	

Many AMPs have broad-spectrum antimicrobial activity against clinical strains of bacteria and fungi. AMPs form secondary structures by electrostatic interactions with bacterial membranes by a salt-sensitive step. Therefore, high salt concentrations in the human body fluids can deactivate many AMPs and it is essential to develop salt-tolerant AMPs for applications in healthcare. However, at physiological condition, pH, temperature and high salt concentrations will influence the activity of these peptides.

We have evaluated the pH and thermostability of Chain201D using radial diffusion assays (RDA). The zones of inhibition for standard conditions (peptide stored at −20 °C, pH 7) were 16 & 15 mm for *E*. *coli* and *S*. *aureus*, respectively, and 15 & 14 mm for *P*. *aeruginosa* and *K*. *pneumonia*, respectively. For the control compound gentamicin, the zones of inhibition were between 22–24 mm. The activity of Chain201D was not affected by changes in pH ranging from 4–9 or storage temperature of even up to 45 °C for over 14 days, as no differences in RDA were observed compared to standard conditions.

The effect of salt concentrations on antimicrobial activity was tested using MIC by adding a range of concentrations (50, 100 and 200 mM) of NaCl to Müller Hinton. Only a 2-fold increase in MIC values of Chain201D was observed upon using 200 mM NaCl concentration.

In a previous publication from our group the control peptide CEM7 was tested against *S*. *aureus* (ATCC 25923) and no bacterial growth inhibition was found using concentrations of up to 512 μg/mL^31^. Previous to immobilization studies, the MIC of CEM7 against *E*. *coli* (ATCC 25922) was also determined and no bacterial growth inhibition was detected using the same concentrations.

### Chain201D model surface

#### Surface Characterization

Proper covalent immobilization of both active peptide (Chain201D) and control peptide (CEM7) on activated SAMs was monitored using X-ray Photoelectron Spectroscopy (XPS), water contact angle (WCA) and ellipsometry. To produce surfaces with varying amounts of immobilized peptides, the OH groups of the EG4-SAMs were first activated using two different concentrations (3 and 30 mg/mL) of CDI (CDI (3) and CDI (30)), and then, a fixed peptide concentration (0.5 mg/mL) was used. We have previously shown that using this strategy, increasing concentrations of peptides/proteins are successfully immobilized on EG4-SAMs^34–36^.

#### XPS

XPS survey spectra revealed the presence of the elements expected in the monolayer and substrate used (Au, C, O, and N), thus indicating the absence of contamination (data not shown). Table 3 presents the relative surface atomic composition (%) calculated from high-resolution XPS spectra of different samples.

**Table 3:** Surface atomic composition (%) calculated from high-resolution XPS spectra of different monolayers. ((−) not detected).

Samples	C1s	N1s	O1s	Au4f	Ratio	
					C/Au	N/Au
EG4	65.9	—	20.6	13.5	4.9	—
CDI (3)	64.9	—	21.9	13.2	4.9	—
CDI (30)	64.4	—	22.2	13.4	4.8	—
Chain201D (3)	67.2	1.6	19.6	11.6	5.8	0.14
Chain201D (30)	68.0	4.5	18.1	9.4	7.2	0.48
CEM7 (3)	67.0	1.7	20.3	11.0	6.1	0.15
CEM7 (30)	67.0	2.2	20.0	10.8	6.2	0.2

Regarding the surface activation with CDI, no changes in surface atomic composition were observed in CDI (3) or CDI (30) compared to the original EG4-SAM, except for a small oxygen increase (Table 3). The absence of nitrogen in these samples is related to their immersion in the buffer, which promotes the release of the imidazole group as previously described by us^37^. Peptide immobilization was easily confirmed by the appearance of nitrogen on peptide-tethered surfaces (Chain201D and CEM7) (Table 3). Indeed, the increase of both ratios N/Au and C/Au reveal an increase of tethered peptide with increasing CDI concentrations. This observation was more evident for SAMs containing Chain201D where N/Au increased from 0.14 to 0.48 and C/Au from 5.8 to 7.2 for CDI (3) and CDI (30), respectively. High-resolution spectra of C1s (data not shown) show expected alterations of the carbon environments upon successive modifications of the surface^37^.

#### Water contact angle measurements (WCA)

**Figure 2:**
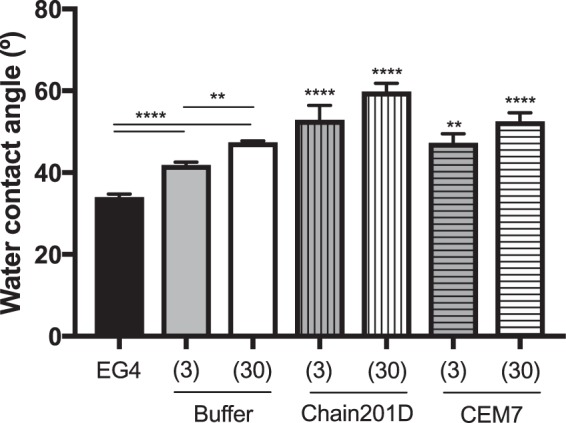
Water contact angle of EG4-SAMs before and after (i) activation with CDI and (ii) peptide immobilization. One-way ANOVA ****p < 0.0001, **p < 0.003.

Results from WCA measurements are depicted in Fig. 2. WCA of both CDI activated EG4-SAMs, CDI (3) and CDI (30), were significantly increased regarding the original EG4-SAMs samples (from 34 ± 0.7° to 42 ± 0.6° and 47 ± 0.3°, respectively), suggesting EG4 functionalization with CDI. After peptide immobilization, SAMs presented a subsequent WCA augmentation, which was more dramatic for immobilized Chain201D samples (Chain201D (3): 53 ± 4° and Chain201D (30): 60 ± 2°) than for control CEM7 samples (CEM7(3): 47 ± 2° and CEM7(30): 53 ± 2°). Moreover, a similar WCA increase was observed between Chain201D (3) and (30) and between CEM7 (3) and (30), which could be related to growing peptide density present on these surfaces.

#### Ellipsometry

**Figure 3:**
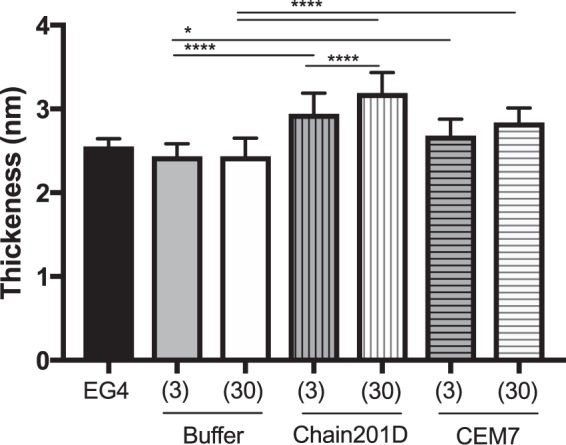
Ellipsometry measurements of modified EG4-SAMs (activated SAMs: CDI (3), CDI (30); and peptide immobilized SAMs: Chain201D and CEM7). Non-parametric Kruskal-Wallis, ****p < 0.0001; *p < 0.05.

Figure 3 shows the monolayer thickness after each step of surface functionalization. SAMs activation with CDI did not change the thickness of EG4-SAMs (2.6 ± 0.1 nm), which remained at 2.4 ± 0.2 nm (CDI (3) and CDI (30)). This was expected, as the sample immersion in the buffer promotes the release of the imidazole group, as described before^37^. Peptide introduction resulted in significant thickness increase for both peptides tested. Regarding peptide immobilization on CDI (3), it is possible to observe an increase of ~0.5 nm to Chain201D (3) and of ~0.2 nm to control CEM7 (3). Concerning CDI (30), the peptide immobilization increased the thickness of the monolayer by ~0.8 nm for Chain201D (30) and by ~0.4 nm for CEM7 (30). The increase in average thickness is most likely related to the increased concentration of the immobilized AMPs, rather than reflecting a true layer thickening. This constitutes indirect evidence for the effective immobilization of AMPs to SAMs. Nevertheless, to have an estimation of the peptides surface density on CDI SAMs, the method described by Qin *et al*.^38^ was applied using the following formula:

$${{\rm{N}}}_{{\rm{peptide}}}=[{\rm{\Delta }}t\times {\rm{\rho }}\times {{\rm{N}}}_{{\rm{A}}}\times {10}^{-8}]/{{\rm{Mw}}}_{{\rm{peptide}}}$$

where Δt is the thickness increase in Å, N is Avogadro’s number (6.02 × 10^23^ molecules/mol), Mw is the peptide molecular weight (Chain201D:1661.01 g/mol and CEM7: 1379.73 g/mol), and ρ is the density of peptide, estimated to be 1.54 g/mL using the following formula proposed by Fischer *et al*.^39^:

$${\rm{\rho }}=[1.41+0.145\,\exp (\,-\,{{\rm{MW}}}_{{\rm{peptide}}}({\rm{kDa}})/13)]=1.54\,{g/\mathrm{cm}}^{3}$$

Results are summarized in Table 4.

**Table 4:** Peptide surface density estimation from monolayer thickness determined using ellipsometry.

	Peptide surface density estimation (ng/cm^2^)	
	Chain201D	CEM7
**CDI (3)**	77	31
**CDI (30)**	123	62

The sources of error include the ellipsometric measurement and the estimation of the peptide weight density (formula 2 was proposed for crystals of proteins with a molecular weight higher than 7 kDa)^38^.

In general, all surface characterization techniques demonstrated that peptides were successfully immobilized onto EG4-SAMs and their surface density was increased with the CDI concentration. Nevertheless, although similar chemistry was used for both peptides, Chain201D immobilization efficiency was much higher (~double) than that of CEM7.

#### Antimicrobial activity of Chain201D model surface

The antimicrobial activity of Chain201D and CEM7 functionalized surfaces was tested simultaneously using *E*. *coli* and *S*. *aureus*. After incubation with bacteria, surfaces were stained with the LIVE/DEAD® Bacterial Viability Kit (Baclight^TM^), or sonicated and plated for colony forming units (CFU) counting. Quantification of non-adherent *E*. *coli* has also been performed by CFU counting and no differences between samples were detected (supplementary material).

**Figure 4:**
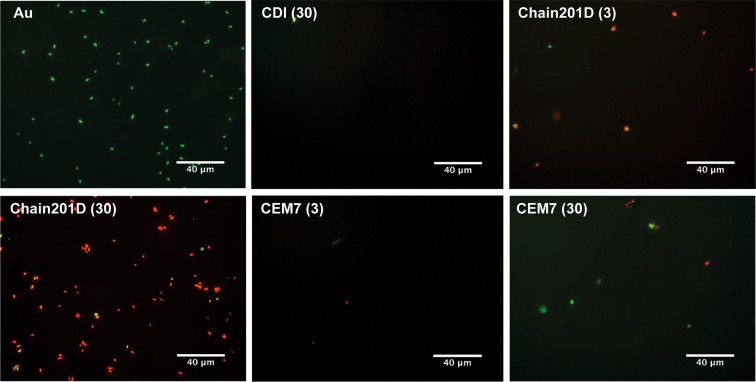
Representative images of EG4-modified SAMs labeled with Live/Dead staining after contact with *E*. *coli*. Images were collected using an inverted fluorescence microscope with 400x magnification.

**Figure 5:**
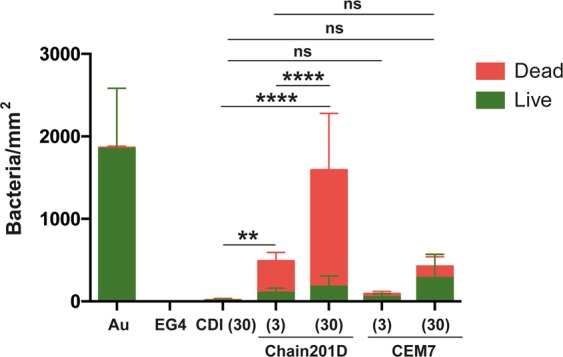
Antimicrobial activity of Chain201D and CEM7 tethered to EG4-SAMs against *E*. *coli*. The viability of surface-adherent bacteria was assessed using Live/Dead staining. Results are the average ± SD of fifteen images. Statistics are presented only for the dead group (One way-ANOVA, correction for multiple comparisons: Sidak test, ****p < 0.0001, **p = 0.0011).

**Figure 6:**
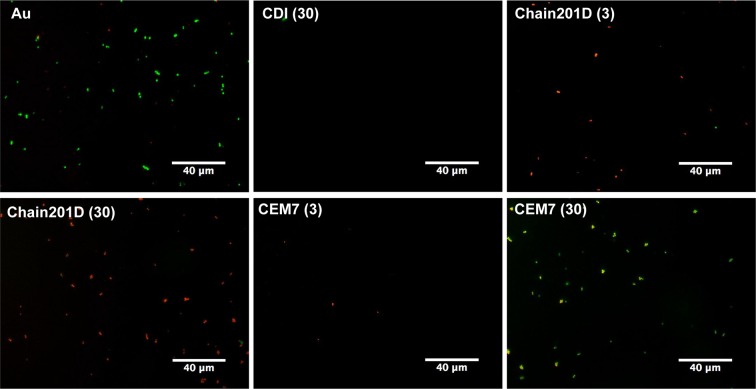
Representative images of EG4-modified SAMs labeled with Live/Dead staining after contact with *S*. *aureus*. Images were collected using an inverted fluorescence microscope with 400x magnification.

**Figure 7:**
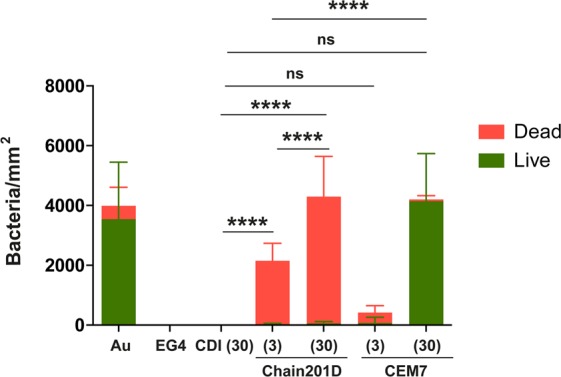
Antimicrobial activity of Chain201D and CEM7 tethered to EG4-SAMs against *S*. *auerus*. The viability of surface-adherent bacteria was assessed using Live/Dead staining. Results are the average ± SD of fifteen images. Statistics are presented only for the dead group (One way-ANOVA, correction for multiple comparisons: Sidak test, ****p < 0.0001).

Live/Dead staining is a combination of two nucleic acid stains SYTO 9 and propidium iodide (PI). SYTO 9 stains all bacteria, while PI penetrates only bacteria with damaged membranes, quenching the signal of SYTO 9 and staining the bacteria in red. Representative images and quantification of adhered *E*. *coli* are shown in Figs 4 and 5, and representative images and quantification of adhered *S*. *aureus* are shown in Figs 6 and 7 respectively. For *E*. *coli*, Chain201D-functionalized surface showed an increased number of adherent bacteria when compared to the control samples EG4 and CDI (30), where bacterial adherence was minimal. EG4 anti-adhesive behaviour is an expected result, and this model surface was chosen to avoid non-specific bacterial adhesion due to its well-known non-fouling properties that easily reveal peptide effects^40^. Moreover, the increase in bacterial adherence to Chain201D surfaces seems to correlate with AMP density, which is about two-fold more in Chain201D (30) than in Chain201D (3) surface (Table 4). Evaluation of the viability of adherent bacteria revealed a high killing percentage on the Chain201D samples, 79% in Chain201D (3) and 89% in Chain201D (30), proving the high antimicrobial efficiency of the surface. For *S*. *aureus*, a similar pattern of adherence and killing was observed, Chain201D-functionalized surface showed an increased number of adherent bacteria when compared to the control samples EG4 and CDI (30). The increase in bacterial adherence to Chain201D (3) and Chain201D (30) surfaces also correlates with AMP density, in the same way as for *E*. *coli* (Table 4). Evaluation of the viability of adherent bacteria revealed a high killing percentage of 99% on the Chain201D (3) and (30) surfaces.

Surfaces functionalized with CEM7 were used as controls, as despite having similar length, positive charge and hydrophobicity, CEM7 does not show antimicrobial activity in solution against *E*. *coli*, *S*. *aureus* or other bacterial strains as published elsewhere^31^. Bacterial adhesion to CEM7 surfaces showed a different pattern for *E*. *coli* and *S*. *aureus*. However most of the bacteria adherent to these surfaces remained viable. While on CEM7 (3) bacterial adherence was minimal for both *E*. *coli* and *S*. *aureus*, on CEM7 (30) adherence of *S*. *aureus* was higher than for *E*. *coli*. For *E*. *coli* adherence to CEM7 (30) was similar to Chain201D (3) surface. This could be related to the fact that CEM7 (30) seems to have a similar peptide density than Chain201D (3) (Table 4). Nevertheless, despite having the same ability to bind bacteria, killing rates were higher in Chain201D than in CEM7-modified surfaces, reaching 79% of dead adherent bacteria in the Chain201D (3) modified surface and only 33% in the CEM7 (30) (Figs 4 and 5).

**Figure 8:**
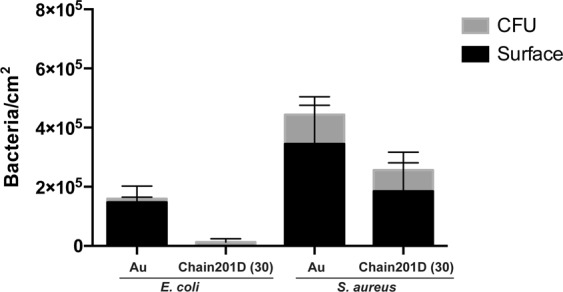
Antimicrobial activity of Chain201D and CEM7 tethered to EG4-SAMs against *E*. *coli* and *S*. *aureus*. Surfaces were sonicated after contact with bacteria and CFU counting was performed to quantify viable detached bacteria (results are the average ± SD of three replicates) while quantification of remaining bacteria on the surface was done by microscopy after DAPI staining. Results are the average ± SD of fifteen images.

**Figure 9:**
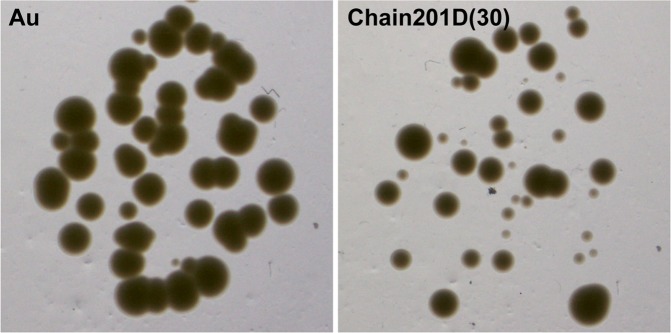
Images of *S*. *aureus* colonies grown on TSA after recovery from Chain201D EG4-SAMs.

Surfaces were also evaluated using sonication to access the number of viable adherent bacteria. CFU counting and quantification of remaining adherent bacteria on the surfaces using DAPI staining was performed (Fig. 8). Under the conditions used *E*. *coli* was more easily released from the surface modified with Chain201D than from the control Au, as no bacterial cells were observed on the surface after sonication. Considering CFU counts (viable bacteria) and the surface observation after sonication, we can conclude that Chain201D surface kills almost all adherent *E*. *coli*. For *S*. *aureus*, approximately half of the bacteria remained attached to the Chain201D modified surface after sonication, when comparing to the total number of adherent bacteria determined in the Live/Dead assay and a small percentage of bacteria released were viable. Therefore for *S*. *aureus*, using this assay, conclusions about the percentage of killing rates cannot be inferred. However, as shown in Fig. 9, *S*. *aureus* bacterial cells recovered from the Chain201D surface do not seem to grow very wells on agar plates, displaying colonies very small in size, which is probably a result of growth impairment due to membrane damage.

## Discussion

In this work we show that the *de novo* -designed AMP Chain201D is a broad-spectrum AMP active against bacteria and yeast, namely on strains relevant in the context of catheter-associated infection. Moreover, Chain201D is stable in a wide range of temperatures and pH and it is salt tolerant, being an excellent candidate for the treatment of catheter-associated infections. Despite its excellent properties, Chain201D application *in vivo* may induce toxicity, as such catheter surface modification appears as an excellent strategy to prevent systemic treatment. Chain201D was tested for antimicrobial activity when coupled on SAMs of alkanethiols on gold, a model surface which allows the covalent immobilization of biomolecules and a surface control at the molecular level, being ideal for studying specific bacteria–ligand interactions^41,42^. An EG4-terminated SAM was chosen to avoid non-specific protein adsorption, guaranteeing specific bioactivity of the immobilized biomolecules. Moreover, a non-active peptide (CEM7) with similar chemical features (similar length, similar positive charge, and a similar number of hydrophobic residues)^31^ was selected as control.

Chain201D maintains its antimicrobial activity when covalently grafted to EG4 SAMs, having a high capacity to bind and kill adherent *E*. *coli* and *S*. *aureus*.

Peptide and protein covalent immobilization using CDI has widely been employed by our group^34,36,37^. This compound converts the free hydroxyl groups of the EG4-SAMs into imidazolyl-carbamate groups, which react with N-nucleophiles (e.g. peptide’s free amine groups) in alkaline conditions^35,37^. Unreacted imidazole groups are removed by hydrolysis at alkaline pH, favoring a clean immobilization^37^. To obtain surfaces with increasing amounts of the immobilized peptide, EG4-SAMs were activated with two different concentrations of CDI (3, and 30 mg/mL) (CDI (3) and CDI (30)) using a fixed peptide concentration^34,36,37,43^. The maximum CDI concentration of 30 mg/mL was selected since it was demonstrated that EG4 activation with 60 mg/mL of CDI does not increase the amount of immobilized peptide^37^.

Evidence of proper EG4-SAMs activation with CDI was found using WCA, by the growing increase in hydrophobicity with the increase of CDI concentration, which is in congruence to the previously reported studies by us^34,37,43,44^. Concerning peptide immobilization, all surface characterization techniques used contributed to corroborate their covalent immobilization in increasing concentrations. The increase of further hydrophobicity (WCA), thickness (ellipsometry) and N/Au ratios (XPS) of the CDI-activated SAMs after immersion in peptide solutions suggest that peptide immobilization has occurred at concentrations that are dependent on the activation degree. However, results also suggested a higher amount of immobilized Chain201D than CEM7. A possible explanation for this observation may be the conformational behavior of each peptide in an alkaline buffer. Indeed, although Chain201D and CEM7 possess similar chemical features, different amino acid composition/sequence may promote the preferable co-precipitation of CEM7, which may reduce the immobilization yield. Nevertheless, all characterization techniques, ellipsometry, WCA and XPS suggest similar peptide concentration on Chain201D (3) and CEM7 (30), which was also corroborated by the similar adhesion of *E*. *coli* observed on these two surfaces. The cationic character of both peptides is probably responsible for the establishment of electrostatic interactions with the negatively charged bacterial membranes, leading to bacterial adhesion to the surfaces^45^. However, despite having the similar ability to attract *E*. *coli* to the surface, the bactericidal effect is much stronger in the presence of Chain201D than that of CEM7, reaching in the case of *E*. *coli* about 80% of dead adherent bacteria in Chain201D in comparison to ~30% in CEM7, according to Live/Dead staining results. Regarding *S*. *aureus*, the correlation between Chain201D and CEM7 concentrations and adhesion does not seem to be so evident. Nevertheless, according to Live/Dead staining, Chain201D surface is very active against *S*. *aureus*, killing 99% of adherent bacteria, while for CEM7 most of the adherent bacteria are alive.

The bactericidal potential of immobilized Chain201D against *E*. *coli* and *S*. *aureus* can be improved by increasing the peptide concentration, since in the case of *E*. *coli* Chain201D (30) can attract, bind, and kill around three times more bacteria than Chain201D (3), although with the same killing percentage (~80%). While for *S*. *aureus*, Chain201D (30) binds, and kills around two times more bacteria than Chain201D (3), with killing percentages of (~99%).

Live/Dead stainings are a very efficient tool to analyse adherence and viability of bacteria on surfaces. However, alterations in bacterial membrane permeability that do not necessarily kill bacteria may give rise to false killed bacterial cells, stained in red^46^. As such, surfaces were also sonicated and plated for CFU counting, and quantification of remaining bacteria on the surfaces was performed. This method was not very efficient to recover bacterial cells from the gold surfaces, as the counts of remaining adherent bacteria were very similar to the total adhesion numbers observed in the Live/Dead assays, and only a small percentage of CFU counts, corresponding to viable released bacterial cells was detected. Nevertheless, this method was more efficient to recover bacterial cells from the Chain201D surfaces, as no *E*. *coli* cells were detected on the surface after sonication, while about half of *S*. *aureus* cells were recovered from the surface when comparing to Live/Dead counts. Regarding *E*. *coli*, using the sonication method, a massive decrease in bacterial counts is observed, supporting the results obtained in the Live/Dead assay. While for *S*. *aureus* conclusions about Chain201D killing rates cannot be inferred using this method as approximately half of the bacterial cells cannot be recovered from the surface. Nevertheless, *S*. *aureus* CFUs after contact with Chain201D display a specific phenotype, demonstrating that bacteria, despite not being all killed, are affected by the contact with the Chain201D surface, most probably by alterations of membrane permeability, which explains the higher numbers of dead bacteria (red stained) observed in the Live/Dead assay.

Some concerns have been raised about the presence of dead adherent bacteria in the AMP-modified surfaces. However a recent report shows that this may not hamper *in vivo* efficiency. Notably, in Tan *et al*. work^47^, a covalently immobilized AMP showed bactericidal activity with high values of dead adhered bacteria at the surface. This high amount of dead adhered bacteria was not impeditive of successful prevention of perioperative corneal infection in an *in vivo* rabbit keratitis model.

In this study, Chain201D and CEM7 were covalently immobilized by the free amine groups, therefore possibly not displaying a specific orientation on the surface, since immobilization may occur from amine groups of the peptide N-terminus or any Lysine side chains. The fact that Chain201D was bactericidal after such immobilization may suggest that orientation is not a determinant factor for the activity of Chain201D. Although several authors have shown the importance of peptide orientation upon immobilization, others have developed efficient antimicrobial surfaces based on random, not oriented covalent immobilization^14,47–52^. Han *et al*. have shown that surface immobilization of cecropin P1 via C- and N-terminal amino acids result in only a slight difference in the ability to capture and kill bacteria despite having different secondary structures and orientations^48^. Moreover, random immobilization may allow higher yields due to the presence of more immobilization sites. Holmberg *et al*. have shown that immobilization of two peptides with a different number of reactive amine groups (GK7-NH2 and GL13K) results in surfaces with different amounts of the peptide, being more efficient when more immobilization sites are available^49^.

Chain201D immobilization was performed without the use of a spacer, and although it is generally accepted that the use of a long and flexible spacer might be determinant for antimicrobial activity, Chain201D was able to kill about 80% of adherent *E*. *coli* and 99% of adherent *S*. *aureus*, according to Live/Dead staining results, directly linked to the surface^13,53,54^.

The antimicrobial surface obtained with Chain201D (30), containing 123 ng/cm^2^ of immobilized AMP, showed the highest killing efficiency. Few authors have investigated the mechanism of action of immobilized peptides and although some propose a membrane permeabilization mode similar to the soluble AMP, others believe that electrostatic imbalances on the membrane surface (release of e.g. Mg^2+^ and Ca^2+^) may be sufficient to trigger fatal cellular events^55^. This last proposal mechanism is most probably the mechanism of action of short peptides such as Chain201D, as this AMP is far too short to penetrate the bacterial membrane.

Several studies have reported a positive relationship between activity and surface concentration^29,55–57^. Indeed, our results show that higher peptide density can attract more bacteria to the surface and consequently kill more bacteria. Nevertheless, peptide performance does not seem to depend on concentration, as both surfaces, Chain201D (3) and Chain201D (30), having different amounts of peptide immobilized, killed about 80% of the adherent *E*. *coli* and 99% of adherent *S*. *aureus*, which probably indicates that both surfaces act by the same mechanism of action. This result is congruent with Glinel *et al*. work^58^, where the bactericidal capacity of immobilized magainin I was not substantially reduced by the lower concentration of immobilized peptide. It remains to be clarified if this situation is associated (or not) with the use of a minimum threshold concentration of immobilized AMP, as suggested by Dutta *et al*.^51^.

In CEM7 (30) surfaces, a certain percentage of dead *E*. *coli* (~33%) was also observed. This bactericidal effect was not expected since this peptide was not bactericidal in solution even at high concentrations (512 μg/mL). However, this result is congruent with the reports by Holmberg *et al*.^49^ and Lombana *et al*.^50^ where the immobilization of non-active peptides (tested in solution against bacteria), revealed a bactericidal effect of 15–30%. Also, Hilpert *et al*. have reported a peptide, which was inactive in solution and became antimicrobial when tethered to a surface^55^.

Urinary catheters can be made of different polymers such as silicon, latex or polyurethane, being silicon catheters the most widely used. All these polymers are quite inert and their functionalization requires surface activation using plasma, gamma or ultraviolet radiation to introduce polar or ionic groups for interaction with bioactive agents. More specifically, argon plasma treatment followed by *in situ* polymerization of allyl glycidyl ether (AGE) in the presence of UV- radiation may be used on silicon to generate epoxy groups which will subsequently react with the peptide amines^59^. This work has shown the potential of Chain201D when immobilized on surfaces through its amine groups. However, a successful application of Chain201D on urinary catheters will still depend on several factors such as the chemistry used for conjugation and grafting efficiency while low fouling properties must have to be assured.

In our work, we have developed a *de novo* AMP with large spectrum of activity and stable in a wide range of conditions, highly effective when covalently immobilized to a surface, therefore being an excellent candidate for application in the production of coatings for medical devices namely for urinary catheters.

## Conclusions

The *de novo* designed AMP, Chain201D was highly effective against strains relevant in the context of urinary catheter associated infections and stable in a wide range of temperatures, pH and salt concentrations. Chain201D was covalently grafted to a model surface with success in different concentrations, without using a spacer and most probably in a random orientation. Our results show that immobilized Chain201D maintains its antimicrobial activity by killing adhered *E*. *coli* and *S*. *aureus*, independently of the surface peptide density, demonstrating its potential for the development of antimicrobial surfaces namely for application on urinary catheters.

## Experimental Section

### Designing peptide Chain201D

By using rational design techniques, we have designed Chain201D (KWIVWRWRFKR) by replacing various amino acids in the original peptide met11 (NRIVQQRTSSR)^22^. Thus, we have designed and modified Chain201D with tryptophan (W), arginine (R) phenylalanine (F) and lysine (K) at various positions using CAMP database (SVW, RF, ANN and DA algorithms)^60^.

### Synthesis of peptides

Peptide Chain201D (KWIVWRWRFKR) was synthesized by Genscript (Piscataway Township, NJ, USA) as D-enantiomers with > 98% purity. Peptide CEM7 (KKAKKFGKAFVK) was synthesized at Porto Peptide Synthesis Facility (POP-UP) with 94% purity. For MIC and RDA assays, lyophilized peptides were diluted in (0.01% acetic acid and 0.02% BSA) and used immediately, or stored at −80 °C in 100-μL aliquots. For peptide immobilization on SAMs, lyophilized peptides were diluted in water type 1 and used immediately.

### Chain201D antimicrobial activity and stability

#### Microorganisms and Growth conditions

In this study the following ATCC strains were used: *E*. *coli* (ATCC 25922), *S*. *aureus* (ATCC 25923), *K*. *pneumoniae* (ATCC 10031) and *P*. *aeruginosa* (ATCC 27853). Also, five clinical isolates of each of the following species were also tested: *E*. *coli*, *K*. *pneumoniae*, *E*. *cloacae*, *P*. *aeruginosa*, *S*. *pneumoniae*, *A*. *baumannii* and *S*. *aureus*. Moreover, four clinical strains of yeasts including *C*. *albicans*, *C*. *glabrata* and *C*. *parapsilosis* were also used. Bacteria were grown on Muller Hinton Agar (MHA) plates overnight at 37 °C (bacteria spreading) and Muller Hinton Broth (MHB) overnight at 37 °C and 150 rpm (bacteria inoculum). Yeast was grown on Potato dextrose agar (PDA) plates overnight at 30 °C.

#### Minimal Inhibitory Concentration (MIC)

Susceptibility of bacteria and yeasts was determined using a micro-broth dilution assay (CLSI, 2006; Wiegand and co-workers)^61^.

Bacteria were grown overnight on a Müller-Hinton agar plate and diluted to a concentration of 5.0 × 10^5^ colony forming units (CFU)/mL in Müller-Hinton broth (MHB). The bacterial suspension (90 µL) and Chain201D, CEM7 or Gentamicin (reference antibiotic) solutions (10 µL) were added at different concentrations to a 96-well polypropylene microtiter plate (Corning Incorporated, USA), and incubated at 37 °C for 18–24 h. MIC for Yeasts was performed following the same protocol as for bacteria with the exception that yeasts were grown overnight on PDA plates and diluted in RPMI and incubated at 30 °C for 24 hours.

The concentrations of peptides and the reference antibiotic ranged from 0.125 to 512 µg/mL. The MIC was recorded as the lowest concentration which inhibited the visual growth of bacteria.

The effect of NaCl on the antimicrobial activity was tested by adding a range of concentrations (50, 100 and 200 mM) of NaCl to Müller Hinton (MH) medium.

### Radial diffusion assay (RDA Assay)

The 30 ml of 1/10 Muller-Hinton broth (MHB) was supplemented with 1% agarose and 5 × 10^5^ CFU/ml bacterial cells was poured into a single-well Omnitray (Nunc) and overlaid with a TSP 96-well plate. Twenty micrograms of each synthesized Chain201D was tested against various strains of bacteria and plate was incubated overnight at 37 °C and the zone of inhibition were recorded. For thermal stability evaluation peptides were diluted to the concentration of 2 mg/ml in PBS buffer (pH 7.4) and incubated at various temperatures, + 4 °C, 25 °C (RT), 37 °C and 45 °C, while the peptides stored at −20 °C were used as control. After each time interval, 100 µl (2 mg/ml) of peptides were taken and stored at −20 °C, until RDA was performed to determine the antibacterial activity. For pH sensitivity evaluation, the peptides were diluted in different pH (pH 4, 5, 6, 7, 8 & 9) and the antibacterial activity of peptides was tested by RDA.

### Preparation of Chain201D model surface

#### Preparation of EG4-SAMs

Gold substrates (1 × 1 cm) obtained from Instituto de Engenharia de Sistemas e Computadores – Microsistemas e Nanotecnologias, Portugal (INESC-MN), were prepared by deposition of thin layers of chromium (2.3 nm) and gold (37 nm) on silicon wafers as described elsewhere^62^. Immediately before use, gold substrates were washed twice in acetone (Merck), rinsed with ethanol (Merck) and immersed in “piranha” solution (7 parts concentrated H_2_SO_4_ and 3 parts 30% H_2_O_2_) for 5 min (caution: this solution reacts violently with many organic materials and should be handled with care). Substrates were cleaned sequentially with ethanol, water type 2, and ethanol in an ultrasonic bath for 3 min and dried with a gentle stream of argon. Then gold substrates were immersed in the alkanethiol solutions ((1-mercapto-11-undecyl)-tetra(ethylene glycol) (EG4-thiol; SensoPath Technologies) prepared in ethanol (99.8%, Merck) at a final concentration of 0.1 mM. Samples were incubated at room temperature (RT) over a 24-h period in a nitrogen environment and protected from light. After incubation, SAMs were rinsed three times with ethanol in an ultrasonic bath for 2 min and dried with a gentle stream of argon.

#### AMP immobilization on EG4-SAMs

Chain201D and CEM7 immobilization (Fig. 1) was performed after activation of EG4-SAMs with 1,1′-Carbonyldiimidazole (CDI) ( ≥ 97.0%, Sigma-Aldrich) (3 and 30 mg mL^−1^) in anhydrous tetrahydrofuran (THF) for 2 h at RT. Peptide immobilization was performed using peptide solutions of 0.5 mg mL^−1^ in 0.01 M sodium borate (borax), pH 9.15. After incubation and shaking at 40 °C, 100 rpm for 24 h, samples were sequentially washed with 1% sodium dodecyl sulfate in saline phosphate buffer (PBS), PBS, water type 1, and dried with a gentle stream of argon. All solutions were sterilized by filtration before use.

For surface characterization (namely for ellipsometry and water contact angle determination) samples were previously dried in a vacuum oven (Raypa, EV 50) for 1 h at RT.

### Surface characterization

#### X-ray Photoelectron Spectroscopy (XPS)

XPS measurements were performed using a Kratos Axis Ultra HSA (UK) with Vision software for data acquisition and analysis (CEMUP – *Centro de Materiais da Universidade do Porto*). The analysis was carried out with a monochromatic Al Kα X-ray source (1486.7 eV), operating at 15 kV (90 W), in FAT mode (Fixed Analyser Transmission). Survey spectra over a range of 0–1350 eV was collected with an analyzer pass energy of 80 eV. High-resolution C*1s*, O*1s*, N*1s* and Au*4f* spectra were collected with an analyzer pass energy of 40 eV. The analysis was performed with the samples tilted at an angle of 70° (photoelectron emission angle with reference to the surface normal). Data acquisition was performed with a pressure lower than 1 × 10^−6^ Pa, and a charge neutralization system was used. For data analysis, the binding energy (BE) scales were referenced by setting the C*1s* BE to 285.0 eV and all spectra analyses and fitting were done using CasaXPS software.

#### Water Contact Angle (WCA)

Measurements were performed using the sessile drop method with a contact angle measuring system from Data Physics, model OCA 15, equipped with a video CCD-camera and SCA 20 software, as described at^62^. Drops of water type 2 (4 µL) were deposited onto samples surfaces and images were taken every 2 s over 300 s. Droplet profiles were fitted using Young-Laplace formula, to calculate the contact angle. The water contact angle of each sample was calculated by extrapolating the time-dependent curve to zero. Results are the average of two measurements of four independent replicates.

#### Ellipsometry

SAM thickness was measured with an imaging ellipsometer, model EP^3^, from Nanofilm Surface Analysis. This ellipsometer was used in a polarizer-compensator-sample-analyzer (PCSA) mode (null ellipsometry). The light source was a solid-state laser (532 nm). The gold substrate refractive index (n) and extinction coefficient (k) were determined by using a delta and psi spectrum with a variation of the angle between 67° and 75°. A refractive index (n) of 0.7360 and extinction coefficient (k) of 2.6911 was obtained for the gold substrate. Measurements were performed in four regions-of-interest to correct for any instrument misalignment. To determine SAM thickness, the same spectrum was obtained by setting n and k for the organic layer as 1.45 and zero, respectively. Results are the average of two independent measurements of four independent replicates.

### Antimicrobial activity of Chain201D model surface

#### Surface adhesion and viability assays

Antimicrobial activity was evaluated by a modified bacterial adhesion assay initially described by Pallavicini *et al*.^63^. The surfaces were placed in a 24 well suspension plate (SARSTEDT, AG & Co., Germany) and a 5-µL drop of bacterial suspension, adjusted to 1 × 10^8^ CFU/mL in PBS, was placed on the top of each sample. Subsequently, the bacterial suspension was covered with a glass coverslip (13 mm diameter), allowing the formation of a thin film that promotes contact between bacteria and the surface. After incubation at 37 °C for 5 h in a wet environment, 500 µL PBS was added to each well, and up-and-down pipetting was performed until detachment of the coverslip, allowing its removal. Surfaces were washed twice with PBS and once with 0.85% sodium chloride (NaCl, Sigma-Aldrich®) and then processed for Live/Dead staining or for CFU counting after sonication.

#### Live/Dead Staining

After washing surfaces were stained with a combination of two dyes, propidium iodide and syto 9 (Baclight^TM^) for 15 min at RT in the dark, according to manufacturer’s instructions. Finally, the surfaces were mounted in slides using VECTASHIELD^®^ Mounting Medium for microscopy observation. Images were obtained with an Inverted Fluorescence Microscope (Axiovert 200 M, Zeiss, Germany). Five images per sample were acquired with a magnification of 400x and analyzed using ImageJ software. The results are presented as representatives of one of the three independent experiments performed. Data are expressed as mean +/− standard deviation of fifteen images acquired in three independent replicates.

#### CFU counting and surface DAPI staining after sonication

After washing, surfaces were transferred to 5 ml SARSTEDT tubes containing 1 ml of 0.5% Tween 80 in PBS and placed on ice, then sonicated using BactoSonic® from BANDELIN at 160 W for 15 min, placed on ice for 5 min, sonicated again for 15 min and put on ice. After, serial dilutions were done and plated for CFU counting. Data are expressed as mean +/− standard deviation of three independent replicates.

The surfaces were transferred to a 24-well plate and fixed for 20 min using 4% paraformaldehyde in PBS, washed three times with PBS and mounted in slides using VECTASHIELD^®^ Mounting Medium with DAPI for microscopy observation. Images were obtained with an Inverted Fluorescence Microscope (Axiovert 200 M, Zeiss, Germany). Five images per sample were acquired with a magnification of 630x and analyzed using ImageJ software. Data are expressed as mean +/− standard deviation of fifteen images acquired in three independent replicates.

### Statistical methods

For surface characterization, One way-ANOVA was performed followed by Tukey’s post hoc testing. The non-parametric Kruskal–Wallis test was applied when Gaussian distribution was not confirmed. For antimicrobial assays, One way-ANOVA was performed followed by a Sidak’s multiple comparisons test using the GraphPad Prism program. Data are expressed as the mean ± standard deviation (SD) and p values of < 0.05 were considered significant.

## Supplementary information

Supplementary Results

## Data Availability

Data is available upon request.
