# Supplementary material for: Prevention of urinary catheter-associated infections by coating antimicrobial peptides from crowberry endophytes
Source: Sci Rep. 2019 Jul 24;9:10753. doi: 10.1038/s41598-019-47108-5 (PMC6656713; doi:10.1038/s41598-019-47108-5)
Supplement: Supplementary file 1 — Supplementary Results [file 41598_2019_47108_MOESM1_ESM.docx]

**Supplementary material**

**Prevention of urinary catheter-associated infections by coating antimicrobial peptides from crowberry endophytes**

Claudia Monteiro^1,2^, Fabíola Costa^1,2^, Anna Maria Pirttilä^3^, Mysore V. Tejesvi^3,4, *^, M. Cristina L. Martins^1,2,5,*^

^1^ *i3S, Instituto de Investigação e Inovação em Saúde, Universidade do Porto, Portugal*

^2^ *INEB, Instituto de Engenharia Biomédica, Universidade do Porto, Rua Alfredo Allen, 208, 4200-135 Porto, Portugal*

^3^ *Department of Ecology and Genetics, University of Oulu, Oulu, Finland*

^4^ *CHAIN ANTIMICROBIALS ltd, Teknologiantie 2, Oulu 90590, Finland*

^5^ *Instituto de Ciências Biomédicas Abel Salazar, Universidade do Porto, Porto, Portugal*

**^*^Corresponding authors, contributed equally:**

**M. Cristina L. Martins**

*INEB, Instituto de Engenharia Biomédica, Universidade do Porto, Rua Alfredo Allen, 208, 4200-135 Porto, Portugal*

Tel: +351 22 6074982, Fax: +351 22 6094567

e-mail: [cmartins@ineb.up.pt](mailto:cmartins@ineb.up.pt)

**Mysore V. Tejesvi**

*CHAIN ANTIMICROBIALS ltd, Teknologiantie 2, Oulu 90590, Finland*

Tel: +358 406303377

e-mail: [**Tejesvi.Mysore@chainantimicrobials.com**](mailto:Tejesvi.Mysore@chainantimicrobials.com) **/** mvtejesvi@gmail.com


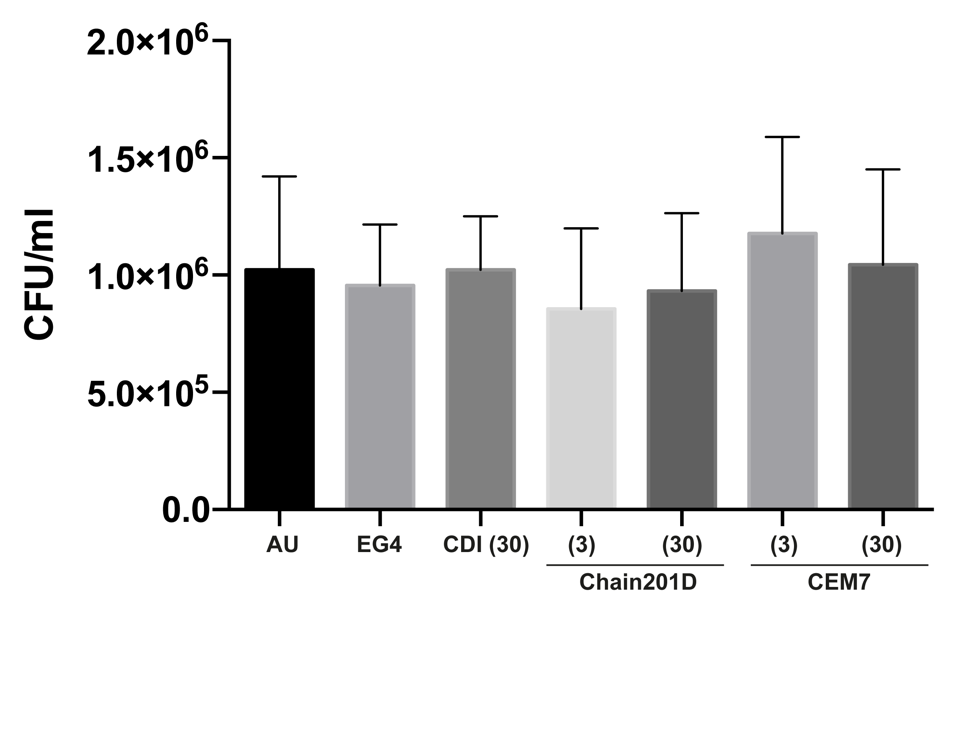


Fig. S1 – Quantification of non-adherent *E. coli* on the **surface** **adhesion and viability assay**. After incubation at 37 °C for 5 h in a wet environment, 500 µL PBS was added to each well, up-and-down pipetting was performed until detachment of the coverslip, allowing its removal. The 500 µL PBS were recovered and serially diluted for CFU counting. Data are expressed as mean +/- standard deviation of three independent replicates. No differences were observed between surfaces.
